# Umbilical cord mesenchymal stem cells protect thymus structure and function in aged C57 mice by downregulating aging-related genes and upregulating autophagy- and anti-oxidative stress-related genes

**DOI:** 10.18632/aging.103594

**Published:** 2020-09-14

**Authors:** Xing-Hua Pan, Qing-Keng Lin, Xiang Yao, Zi-An Li, Xue-Min Cai, Rong-Qing Pang, Guang-Ping Ruan

**Affiliations:** 1 Kunming Key Laboratory of Stem Cell and Regenerative Medicine, 920th Hospital of The PLA Joint Logistics Support Force, Kunming, Yunnan Province, China; 2 Stem Cell and Immune Cell Biomedical Techniques Integrated Engineering Laboratory of States and Regions, Kunming, Yunnan Province, China; 3 Cell Therapy Technology Transfer Medical Key Laboratory of Yunnan Province, Kunming, Yunnan Province, China

**Keywords:** umbilical cord mesenchymal stem cells, senescence, transplantation, thymus, aged

## Abstract

Background: To study the effect of allogeneic umbilical cord mesenchymal stem cell transplantation on the structure and function of the thymus in aged C57 mice and provide a new method for the treatment of senile thymic atrophy.

Results: The changes in the thymus cortex and medulla volume and the lymphocyte ratio were analyzed by immunofluorescence. For thymus tissue sections, immunohistochemical staining was performed to detect p16, p53, SOD, becline1, LC3b, p62, sirt1, and sirt3. Changes in CK5, CK8, CD4 and CD8 expression were observed. Treatment with mUCMSCs could promote hair regeneration in aging mice and regenerate the thymus structure.

Conclusions: mUCMSCs inhibited senescence of the thymus and promoted structural and functional thymus regeneration by downregulating the senescence genes p53 and p16 and upregulating the SOD, Sirt1 and Sirt3 genes, but the mechanism requires further research.

Methods: C57 mice were obtained and met the requirements of thymic aging. mUCMSCs were infused via the tail vein at a dose of 1×10^7^ cells/kg twice per week for 3 weeks. Six weeks after the last transplantation, the thymus was weighed, and the thymus-to-body weight ratio was calculated. The thymus tissue was stained with HE.

## INTRODUCTION

The immune system consists of immune organs, immune cells and immune molecules. Its functions include identifying and excluding antigenic foreign bodies and coordinating with other systems of the body to maintain the stability and physiological balance of the internal environment. As a central immune organ in the human body, the thymus is important for maintaining the stability of the immune system. However, the thymus is age-sensitive and is one of the earliest organs to age and atrophy in the human body. Therefore, whether it is possible to regenerate the thymus to slow its aging and shrinking is an important question.

As a site of T cell development and maturation, the thymus often undergoes atrophy due to aging. The loss of thymocytes and destruction of the thymus structure caused by thymus atrophy reduce mature T cell output and limit T cell receptor (TCR) diversity. Maintaining normal thymic function is critical for reducing the morbidity and mortality of various clinical conditions, including infection and transplantation [1, 2].

The mammalian thymus is generally divided into two leaves, and each leaf is divided into the central medulla and peripheral cortex. The main constituent cells are thymic stromal cells (TSCs), epithelial Mo-MΦ cells, delayed reaction T cells (TDCs), fibroblasts, and thymocytes. The thymus is at its largest and in its most active periods before birth and during puberty. After these periods, it gradually shrinks and is replaced by fat.

The thymus is mainly composed of hematopoietic thymocytes and nonhematopoietic thymic epithelial cells (TECs) [3]. To provide insight into the structure of the thymic microenvironment during thymic aging, to provide a method to maintain regulatory networks and to provide a potential strategy for the recovery of senile thymic function, we searched the literature and found that age-related thymic degeneration is mainly caused by defects in nonhematopoietic TECs, which are characterized by dysfunction of multiple transcription factors (TFs), such as P63 and FOXN1. It also involves other relevant regulatory mechanisms. The involved TFs and regulators are controlled by complex regulatory networks in which microRNAs (miRNAs) are important. miRNAs can directly target the 3' untranslated regions (UTRs) of TFs to inhibit and increase TF expression and control age-related thymic degeneration [4]. After astronauts return from space flight, increasing the thymus potential to reduce thymic atrophy involves reducing the thymus output. T cell function is reconstituted after HIV infection. To provide solutions for senile thymic atrophy, numerous scientific research projects have carried out experimental studies on rodent thymus atrophy and clinical cases of thymic atrophy [5]. The research results are summarized as follows. 1. Zinc supplementation. It has been reported that a diet lacking zinc can cause thymocyte function in mice to be low. The levels of corticosterone in the thymus and spleen of zinc-deficient rats are increased, and the number of T cells is decreased [6, 7]. Low GSH (glutathione) levels are directly associated with poor survival in HIV-infected individuals. Studies have shown that the pre-GSH drug N-acetylcysteine (Nac) increases the GSH level of whole blood CD4+ T cells in HIV patients [8]. In most HIV patients, high-dose combination therapy with Nac and vitamin C results in increased CD4^+^ T cell counts and intracellular GSH levels and decreased plasma levels of HIV RNA [2], which delays the progression of HIV disease. The underlying mechanism may be related to oral antioxidants, and vitamin E can reduce thymus atrophy [9]. 2. Cytokines, chemokines and the lymphocyte factor IL-7 are mainly secreted by nonhematopoietic stromal cells. Although IL-7 has a controversial effect on thymus production in aged mice, it promotes thymus production and peripheral T cell proliferation in young mice. In contrast, IL-7 did not enhance thymus function in aged mice or induce spleen T cell proliferation [10, 11]. 3. Hormones and growth factors. Glucocorticoids (GCs) can directly mediate thymocyte apoptosis. Leptin, growth hormone (GH), keratinocyte growth factor, and other factors can change the balance between thymus atrophy and regeneration to a certain degree [12]. However, although these studies attempted to clarify the mechanism of thymic atrophy, the reliability of the described treatments is not satisfactory.

The thymic microenvironment consists primarily of TECs and cells of mesenchymal origin. TECs are important in almost all stages of thymocyte development. The mesenchyme is involved in the functional development of epithelial cells and the maintenance of epithelial structure and function. The mesenchyme has been shown to be necessary for the successful reconstruction of thymus tissue [13]. Mesenchymal stem cells (MSCs) are a heterogeneous lineage of stromal stem cells that are widely found in many tissues of the fetus and adult and can differentiate into mesoderm and other embryo-derived cells. Bone marrow MSCs have been used to treat a variety of diseases, such as graft versus host disease, systemic lupus erythematosus and rheumatoid arthritis. The mechanisms of action of MSCs in the treatment of these diseases include anti-inflammatory and immunomodulatory effects. In addition, MSCs can also increase the proportion of peripheral blood regulatory T cells (Treg cells) in patients with rheumatoid arthritis. Since Treg cells are mainly produced in the thymus, it is believed that the therapeutic effect of MSCs may be achieved by regulating thymus function. In a previous study, we observed that the size of the thymus in aged mice was increased after MSC transplantation compared with the control treatment over the same period of time. MSC transplantation can promote thymus regeneration.

This study aimed to systematically demonstrate the improvement of senile thymic atrophy induced by administration of MSCs in naturally aging C57 mice to provide a solution for the treatment of clinical thymic atrophy. This study provides additional experimental evidence for the clinical application of MSCs.

## RESULTS

### Appearance and thymus structure changes in 18-month-old C57 mice

### Hair color change in 18-month-old C57 mice

**Figure 1:**
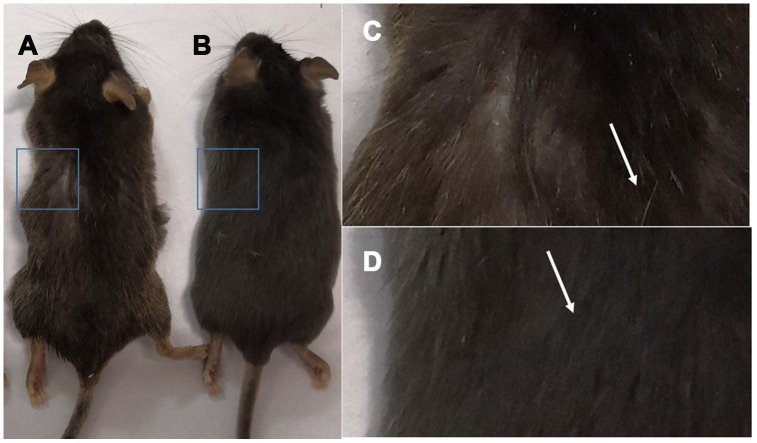
**Comparison of the appearance of C57 mice of different ages.** Note: (**A**) shows 18-month-old C57 mice, and (**B**) shows 2-month-old C57 mice; (**C**) is a photograph of the backs of 18-month-old mice; (**D**) is a photograph of the backs of 2-month-old C57 mice.

Gross view with the naked eye: white hair appeared in C57 mice at 18 months of age (Figure 1C), and the hair of 2-month-old mice was smooth and without white hair (Figure 1D).

### Thymus morphology and size and the thymus index

**Figure 2:**
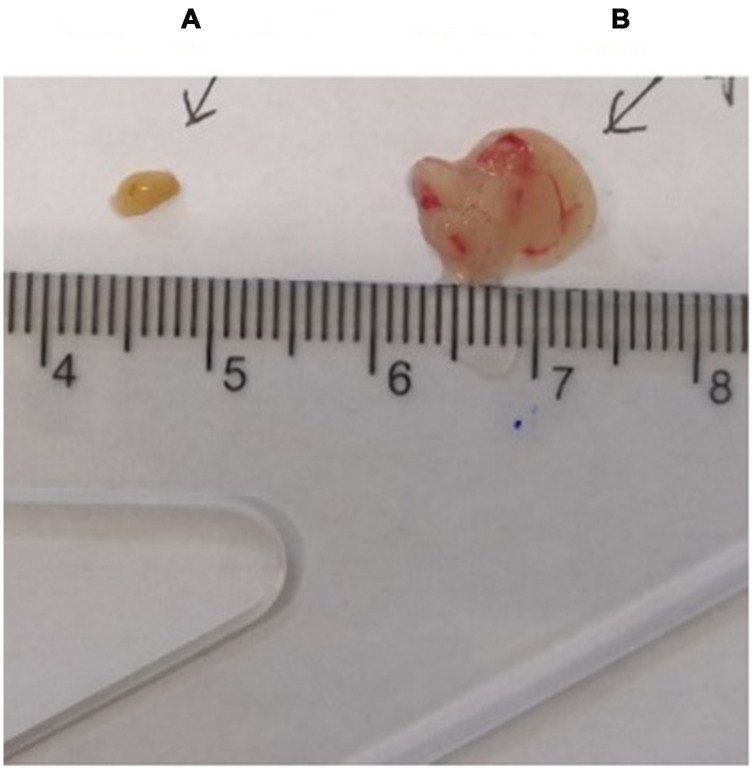
**Comparison of the thymus in C57 mice of different ages.** (**A**) model control group; (**B**) young control group.

The thymus weight, body weight, and thymus/body weight ratio of C57 mice are shown in Table 1; the mouse thymus size was observed visually (Figure 2).

**Table 1:** Comparison of thymus weights in C57 mice of different ages.

	**Thymus weight (mg)**	**body weight (g)**	**Organ index ×10^-3^**
Youth control group	188	29.17	6.444978
Model control group	12	30.7	0.390879

### Thymus tissue structure changes

**Figure 3:**
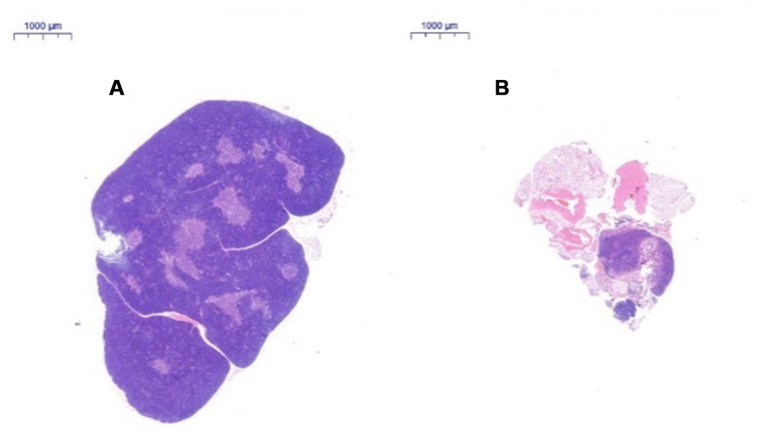
**Changes in the thymus tissue structure in C57 mice of different ages.** Note: (**A**) shows the thymus from a 2-month-old C57 mouse (20×); (**B**) shows the thymus from an 18-month-old C57 mouse (20×). HE staining of the thymus in 18-month-old C57 mice: the volume of the thymus was reduced, and the boundary of the cortex and medulla was not clear; most of the thymus tissue had been replaced by adipose tissue.

After HE staining, the tissue was observed by inverted microscopy. (1) HE staining of the thymus in 2-month-old C57 mice: A thin connective tissue envelope was observed on the surface of the thymus tissue, the thymocyte leaflet interval was not obvious, and the cortex and medulla were clearly defined. The thymus cells in the cortex were dense, and its coloration was deeper. The medulla contained more TECs and had lighter coloration. A large thymus were observed in young mice (Figure 3A). (2) HE staining of the thymus in 18-month-old C57 mice: The volume of the thymus was reduced, and the boundary of the cortex and medulla was not clear; most of the thymus tissue had been replaced by adipose tissue, and only a small portion of the cortex and medulla remained. A small number of lymphoid cells were scattered in the adipose tissue, which is a typical characteristic of old age (Figure 3B).

### Cell growth morphology

**Figure 4:**
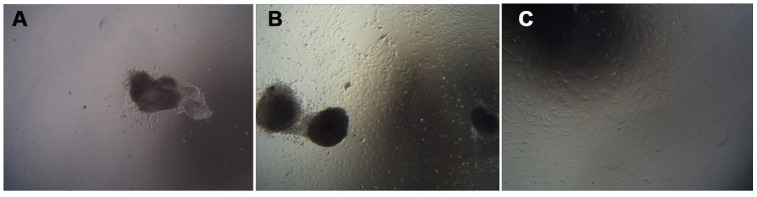
**Cell growth morphology (100×).** Note: (**A**) shows the first day of primary culture, as the cells migrate from the umbilical cord tissue. (**B**) shows the sixth day of primary culture, and the cells that grew were observed around the umbilical cord tissue. (**C**) is a cell from the P3 generation.

The umbilical cord tissue pieces were inoculated in a 10 cm culture dish based on the characteristics of adherent mUCMSC growth. After 1 day, the cells were observed climbing out of the dish (Figure 4A). After 6 days, the cells isolated from the umbilical cord tissue expanded and spread within the surrounding space, and the confluence of the long spindle cells reached 80% (Figure 4B). Passaging was carried out, and the first purification sort was performed. After the P3 generation, the cells were homogeneous, long, fusiform and fibroblast-like (Figure 4C).

### Adipogenic, osteogenic, and chondrogenic differentiation

**Figure 5:**
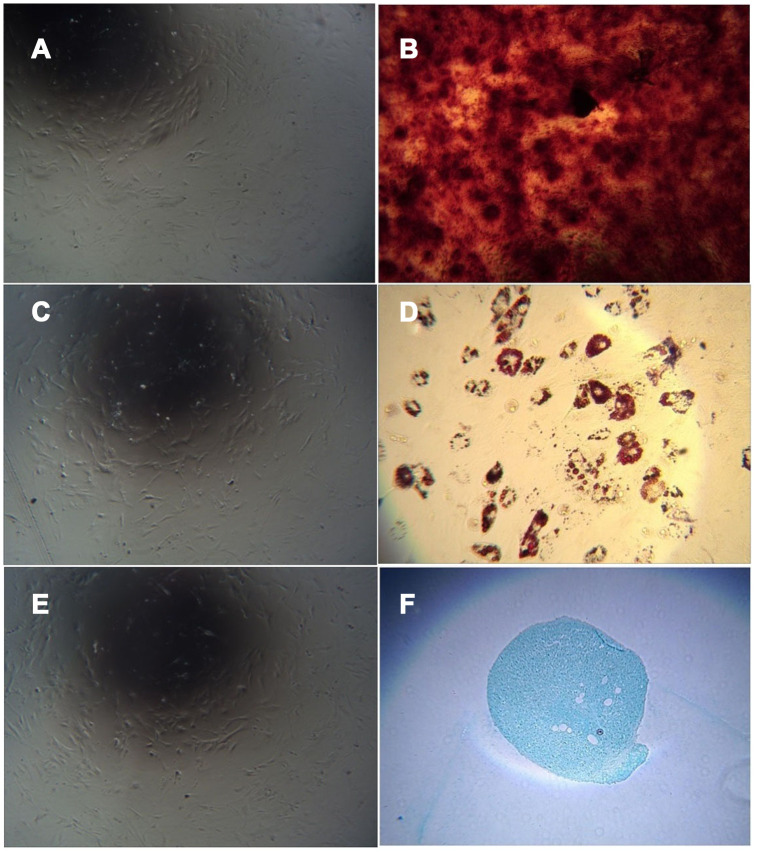
**Cell differentiation induced by adherent culture of C57 mouse umbilical cord tissue.** Note: (**A**) Adherent cells from C57 mouse umbilical cord tissue cultured in mesenchymal medium as a control group; (**B**) Adherent cells from C57 mouse umbilical cord tissue cultured with osteogenic induction of calcium nodules; (**C**) Uninduced cells are not stained; (**D**) Adherent cells from C57 mouse umbilical cord tissue with adipogenic induction after lipid droplets appeared; (**E**) Uninduced cells are not stained. (**F**) Adherent cells from C57 mouse umbilical cord tissue showing the cartilage collagen matrix after cartilage induction.

The cells obtained by adherent culture of umbilical cord tissue were long, spindle-shaped and fibroblast-like (Figure 5A). After culturing in osteogenic induction medium and staining with alizarin red, red-stained calcium nodules were observed (Figure 5B). After culture in adipogenic induction medium, the cells became round, and small lipid droplets appeared in the cells, which were stained with oil red O. Red-stained lipid droplets were visible (Figure 5D). After culture in chondrogenic induction medium, staining with alcian blue caused the cartilage tissue to appear blue (Figure 5F). Uninduced cells were not stained (Figure 5A, 5C, 5E).

### Cellular immunophenotyping

**Figure 6:**
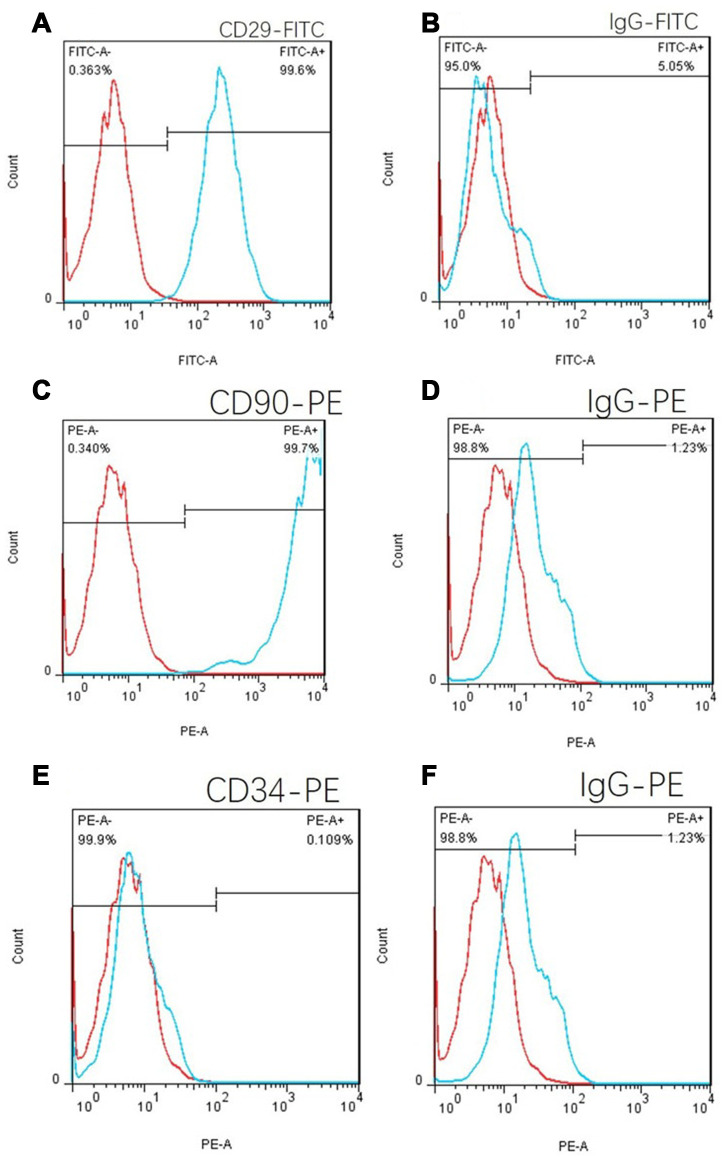
**Cell flow phenotypic results of the adherent culture of umbilical cord tissue in C57 mice.** (**A**) The positive rate of CD29 was 99.6%. (**B**) FITC isotype control. (**C**) The CD90 positive rate was 99.7%. (**E**) The positive rate of CD34 was 0.109%, which is in line with the phenotypic characteristics of mouse mesenchymal stem cells. (**D**) and (**F**): PE isotype control.

CD29, CD90 and CD34 antibodies were used to label C57 mouse umbilical cord tissue adherent culture cells. The positive proportion of CD29 cells was 99.6% (Figure 6A), and the CD90 positive proportion was 99.7% according to flow cytometry (Figure 6C). The positive proportion of CD34 cells was 0.109% (Figure 6E), which was in line with the phenotypic characteristics of mouse MSCs. Isotype controls are Figure 6B, 6D and 6E.

### Hair color change in C57 mice

**Figure 7:**
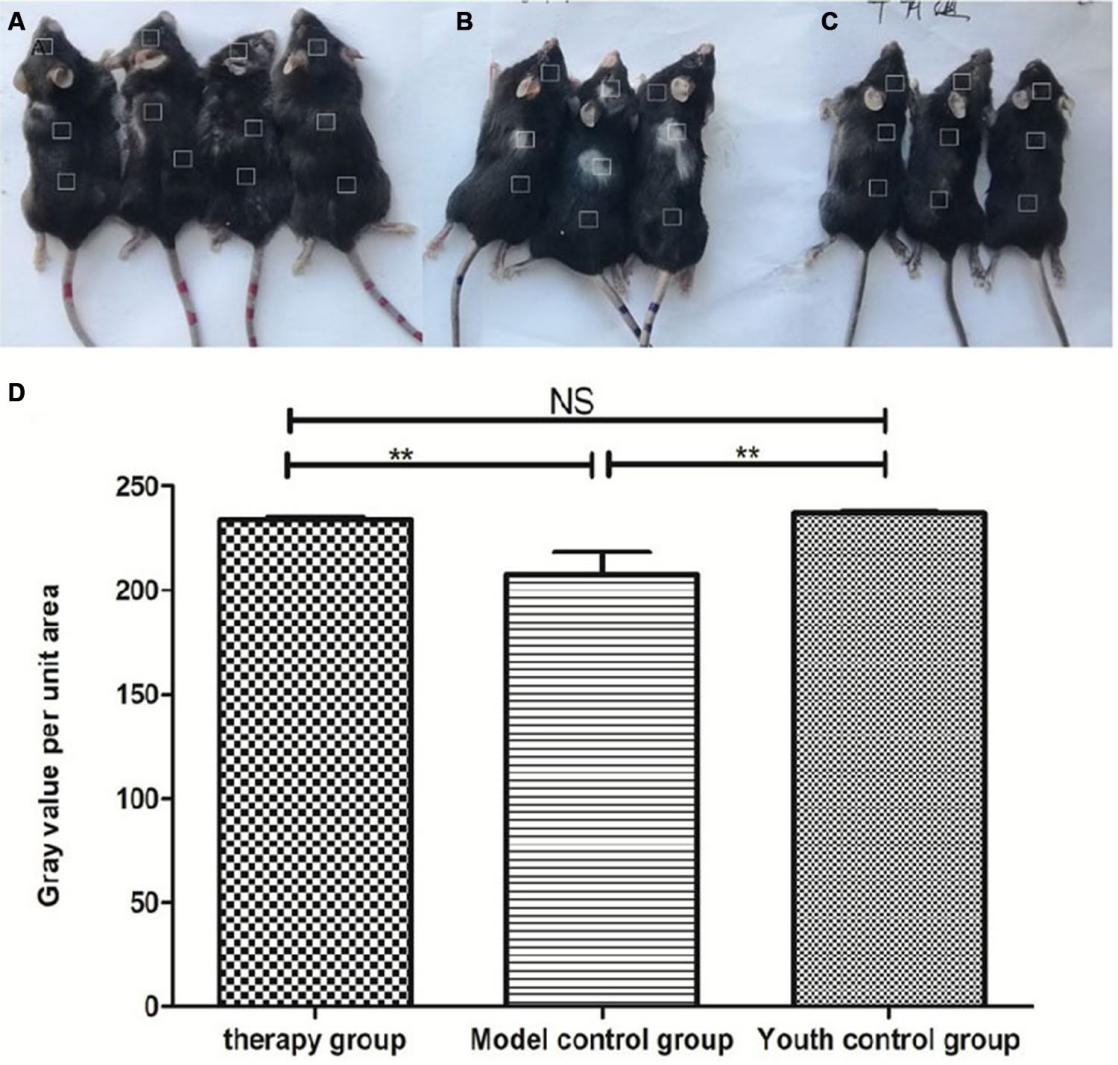
**Comparison of the appearance of mice after mUCMSC treatment.** Note: (**A**) is the treatment group. The hair on the back of the treatment group regrew, and the hair turned black. (**B**) is the model control group. The model control group showed white hair and partial hair loss on the back. (**C**) is the young control group. (**D**) shows the grayscale changes of the mice after mUCMSC treatment; *** indicates p < 0.001; ** indicates p < 0.01; * indicates p <0.05, NS represents p>0.05.

Gross view with the naked eye: The model control group showed white hair and partial hair loss on the back (Figure 7B). The hair on the back of the treatment group regrew, and the hair turned black (Figure 7A). The grayscale values were measured and statistically analyzed with PS software; the grayscale values of the hair of the treatment group were higher than those of the model control group (p<0.01) (Figure 7D, Table 2).

**Table 2:** Comparison of hair grayscale values among the treatment group, the model control group and the young control group on the 28^th^ day after the last cell transplantation (n=10).

	**Number**	**Average grayscale value**		
		**Head**	**Back**	**Hip**
therapy group	1	224.584900	226.486500	237.086000
	2	226.692800	236.065000	240.505800
	3	228.995100	236.873100	241.473300
	4	227.834100	239.082500	237.032800
Model control group ^a^	1	235.332200	174.640200	234.459100
	2	183.543900	176.022000	236.832800
	3	232.655900	162.153500	233.007700
Youth control group ^bc^	1	233.800600	239.135800	235.790600
	2	233.593600	240.373500	239.009100
	3	238.075300	238.796600	236.279800

Note: a is p<0.01 compared with the treatment group; b is p>0.05 compared with the treatment group; c is p<0.01 compared with the model control group.

### Thymus morphology and size and the thymus index

**Figure 8:**
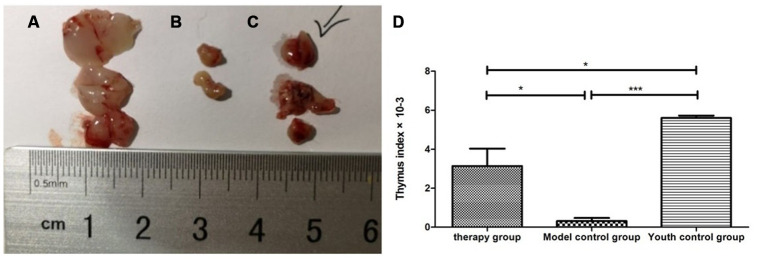
**Changes in the thymus index in mice after mUCMSC treatment.** (**A**) Young control group; (**B**) model control group; (**C**) treatment group; (**D**) thymus index after mUCMSC treatment. According to statistical analysis, the mean values of the thymus index in the young group, the treatment group, and the model control group were 5.60 ± 0.21, 3.10 ± 1.54, and 0.47 ± 0.21, respectively.

Gross view with the naked eye: The thymus in the young control group was yellowish in color, soft in texture and normal in size. The thymus color in the treatment group and the model control group was darker, and the volume was reduced. The volume of the treatment group was significantly larger than that of the model control group (Figure 8). According to statistical analysis, the mean values of the thymus index in the young group, the treatment group, and the model control group were 5.60 ± 0.21, 3.10 ± 1.54, and 0.47 ± 0.21, respectively (Table 3).

**Table 3:** Comparison of the thymus index among the treatment group, the model control group and the young control group on the 28^th^ day after the last cell transplantation (n=10).

	**Number**	**Thymus weight (mg)**	**body weight (g)_**	**Organ index ×10^-3^**
Therapy group	1	72	31.40	2.292994
	2	67	30.51	2.196001
	3	147	29.90	4.916388
Model control group	1	16	35.60	0.449438^a^
	2	20	40.50	0.493827^a^
	3	0	39.80	0 ^a^
Youth control group	1	143	24.80	5.766129^bc^
	2	137	25.60	5.351563^bc^
	3	137	24.10	5.684647^bc^

Note: a is p<0.05 compared with the treatment group; b is p<0.05 compared with the treatment group; c is p<0.001 compared with the model control group.

### Changes in the thymus tissue structure after mUCMSC treatment

**Figure 9:**
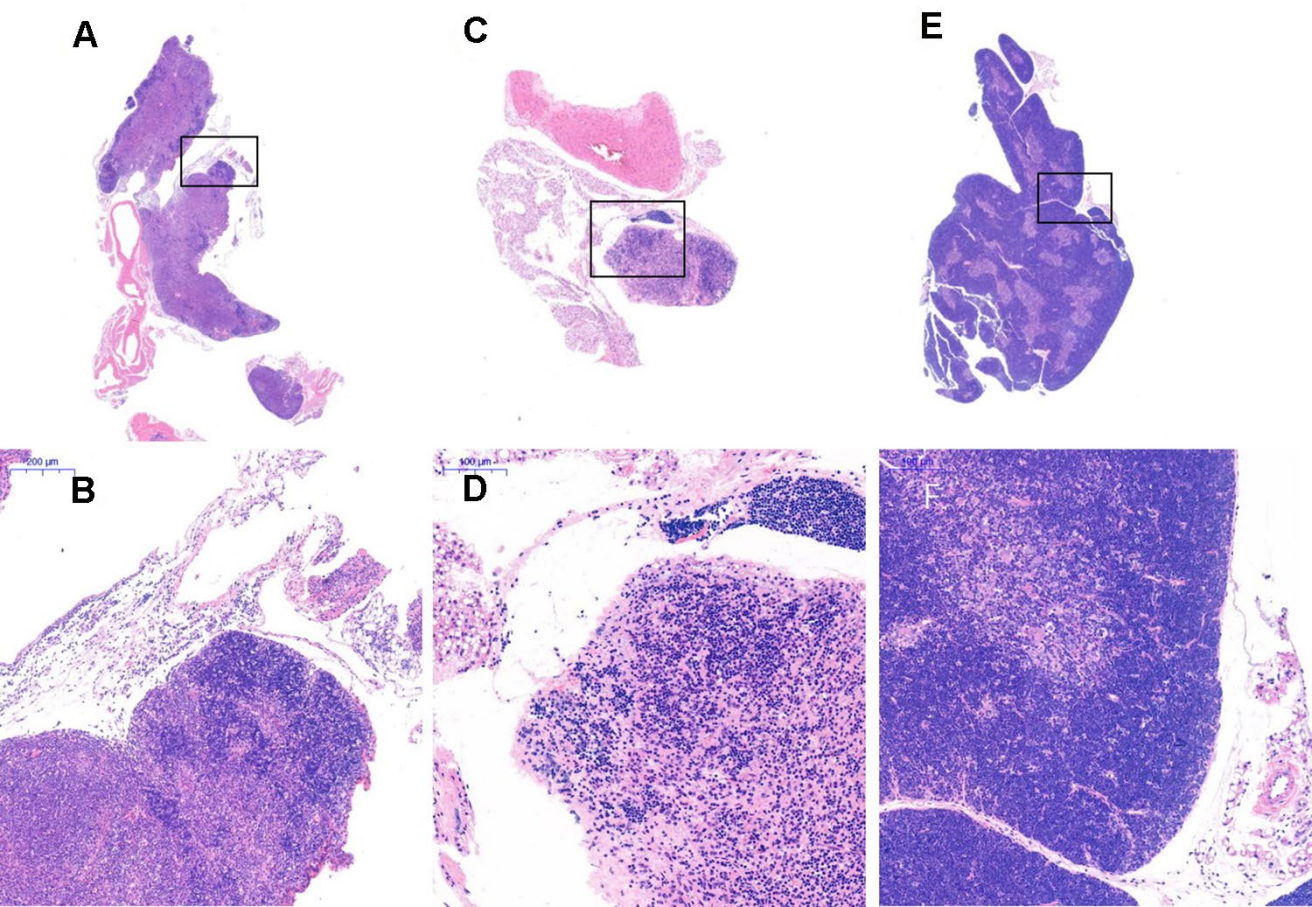
**Thymus tissue structure changes.** Note: (**A**, **B**) show the treatment group after 1 month (20×, 200×); (**C**, **D**) show the model control group (20×, 200×). The thymus tissue of the model control group was reduced in size and unclear; (**E**, **F**) show the young control group (20×, 200×).

After HE staining, an inverted microscope was used to make observations. (1) The thin layer of connective tissue was visible on the surface of the thymus tissue of the young control group. The thymocyte leaflet interval was not obvious, the cortex and medulla were clearly defined, and the thymus cells in the cortex were dense, so the coloration was deep. The medulla contained more TECs, so the coloration was lighter, and the appearance of the thymus was consistent with that observed in young mice (Figure 9A, 9B). (2) The thymus tissue in the treatment group was reduced in size and did not have the shape of small leaves; the cortical portion was greatly reduced, and the boundary of the cortex and medulla was not clear. Part of the medulla appeared to be apoptotic and necrotic, and the nuclei were deeply stained; the cytoplasmic eosinophils were enhanced (Figure 9C, 9D). (3) The thymus tissue of the model control group was reduced in size and unclear. Only a few diffusely distributed lymphoid cells and a small amount of connective tissue were observed (Figure 9E, 9F).

### Expression changes in CK8 and CK5 in thymus tissue

**Figure 10:**
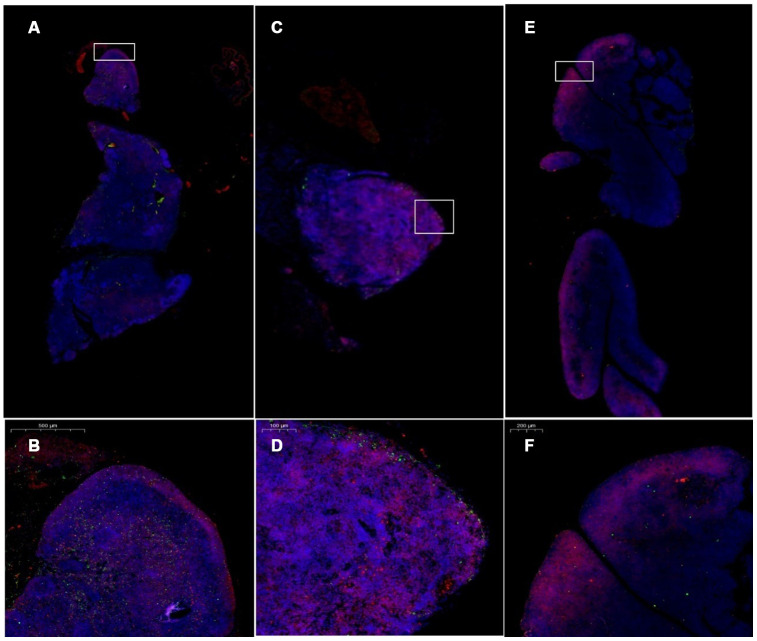
**Changes in CK5 and CK8 expression in the thymus after treatment with mUCMSCs.** Note: (**A**, **B**) are immunofluorescence staining images of thymus tissue in mice treated with mUCMSCs for 1 month; (**C**, **D**) are immunofluorescence staining images of mouse thymus tissue from the model control group; (**E**, **F**) show the thymus in the young control group. Immunofluorescence staining map. CK5 is red, and CK8 is green. Note: *** indicates p < 0.001; ** indicates p < 0.01; * indicates p < 0.05; NS is p > 0.05. Cytokeratin 8 (cK8) and cytokeratin 5 (cK5) are markers of the thymic cortex and medulla, respectively, as shown by the results of thymic staining with immunofluorescent antibodies.

**Figure 11:**
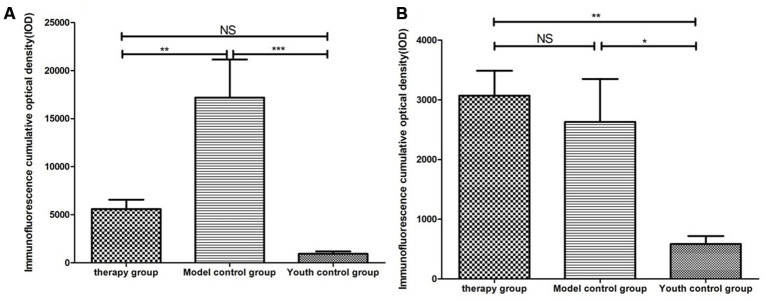
**Differential expression of CK5 and CK8 in the thymus in different groups.** (**A**) Thymus cytokeratin 5 expression after mUCMSC treatment; (**B**) Thymus cytokeratin 8 expression after mUCMSC treatment. The expression of CK5 in the thymus tissue of the treatment group was higher than that in the model control group.

Cytokeratin 8 (cK8) and cytokeratin 5 (cK5) are markers of the thymic cortex and medulla, respectively, as shown by the results of thymus staining with immunofluorescent antibodies (Figure 10). (1) The expression levels of CK5 and CK8 in the thymus of the model control group and the treatment group were higher than those in the young group (p<0.01). (2) The expression of CK5 in the thymus tissue of the treatment group was higher than that in the model control group (p<0.01); the expression level of CK8 was increased, but the difference was not statistically significant (p>0.05) (Figure 11, Table 4).

**Table 4:** Comparison of CK5 and CK8 expression levels among the treatment group, the model control group and the young control group after mUCMSC treatment (n=9).

**CK5 expression level**	**therapy group**			**Model control group**			**Youth control group**		
Number	1	2	3	1	2	3	1	2	3
Immunofluorescence cumulative optical density (IOD)	10147.4	4790.3	6488.0	21098.2^a^	30361.8^a^	20042.8^a^	282.6^bc^	2593.0^bc^	742.8^bc^
	5228.96	5174.4	2568.0	14635.5^a^	38061.2^a^	17175.0^a^	647.1^bc^	1468.4^bc^	656.7^bc^
	1533.5	10049.5	4363.8	2596.02^a^	5596.16^a^	5200.71^a^	476.5^bc^	608.59^bc^	973.0^bc^
CK8 expression level	1	2	3	1	2	3	1	2	3
Immunofluorescence cumulative optical density (IOD)	5475.67	2682.4	2004.5	1844.08^d^	4408.2^d^	3671.7^d^	314.2^ef^	1500.2^ef^	299.5^ef^
	2213.64	4645.1	2100.0	1658.94^d^	7315.7^d^	1716.1^d^	418.4^ef^	760.8^ef^	674.3^ef^
	2305.85	3692.9	2491.4	298.94^d^	1107.2^d^	1645.0^d^	185.7^ef^	739.0^ef^	374.0^ef^

Note: a is p<0.01 compared with the treatment group; b is p>0.05 compared with the treatment group; c is p<0.001 compared with the model control group. d is p>0.05 compared with the treatment group; e is p<0.01 compared with the treatment group; f is p<0.05 compared with the model control group.

### Expression of CD4 and CD8 in the thymus after treatment with mUCMSCs

**Figure 12:**
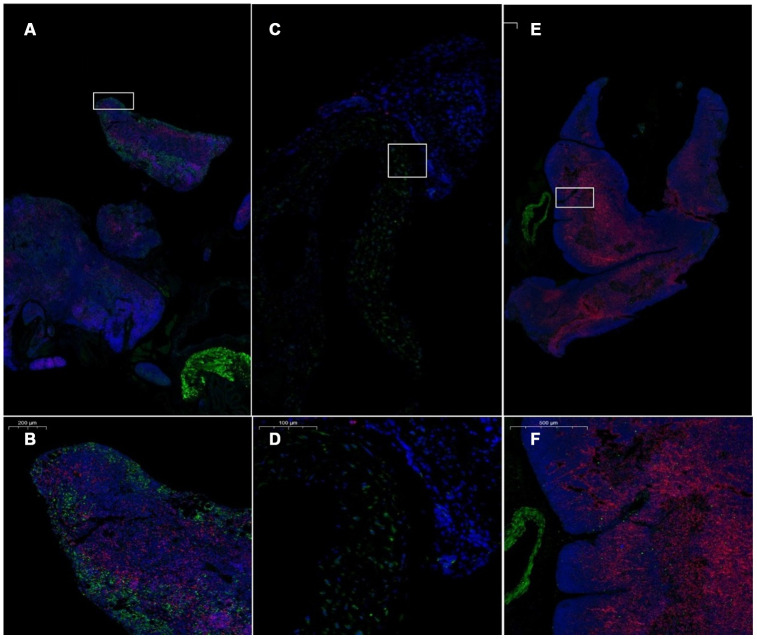
**Pathological morphology of the thymus after transplantation of umbilical cord mesenchymal stem cells.** Note: (**A**, **B**) show immunofluorescence staining of thymus tissue CD4 and CD8 cells in the treatment group. (**C**, **D**) show immunofluorescence staining of CD4 and CD8 cells in the thymus tissue of model control mice. (**E**, **F**) show immunofluorescence staining of CD4 and CD8 cells in the thymus tissue of young control mice. CD4 is red, and CD8 is green. The number of CD4^+^ and CD8^+^ cells in the thymus of the model control mice was small.

**Figure 13:**
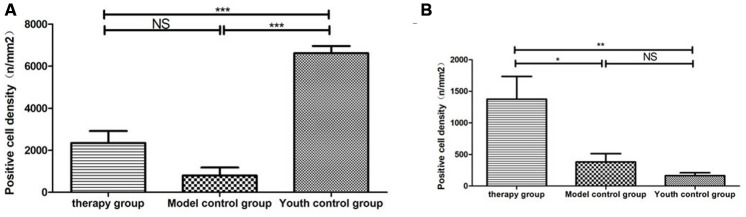
**Differential expression of CD4 and CD8 in the thymus between different groups.** (**A**) Thymus CD4 expression after mUCMSC treatment; (**B**) Thymus CD8 expression after mUCMSC treatment. CD4^+^ lymphocytes were predominant in the young control group, and CD8^+^ lymphocytes were rare. Note *** indicates p value <0.001, ** indicates p value <0.01, * indicates p value <0.05, NS indicates p value>0.05.

The number of CD4^+^ and CD8^+^ cells in the thymus of the model control mice was low (Figure 12). CD4^+^ and CD8^+^ cells in the thymus of the mice in the treatment group reappeared in the plastid structure. The increase in CD8^+^ cells was most pronounced. CD4^+^ lymphocytes were predominant in the young control group, and CD8^+^ lymphocytes were rare (Figure 13, Table 5).

**Table 5:** Comparison of the CD4 and CD8 expression levels among the treatment group, the model control group and the young control group after mUCMSC treatment (n=9).

**CD4 expression level**	**therapy group**			**Model control group**			**Youth control group**		
Number	1	2	3	1	2	3	1	2	3
Immunofluorescence cumulative optical density (IOD)	3557.3	5108.0	544.85	1451.2^a^	0^a^	62.86^a^	5972.4^bc^	7245.5^bc^	6124.4^bc^
	3295.3	3222.0	371.97	2315.6^a^	0^a^	20.95^a^	6664.0^bc^	6234.4^bc^	7318.9^bc^
	3274.3	1346.4	408.6	916.8^a^	0^a^	5.23^a^	4688.9^bc^	7041.2^bc^	8272.4^bc^
CD8 expression level	1	2	3	1	2	3	1	2	3
Immunofluorescence cumulative optical density (IOD)	1922.7	2148.0	83.8	633.9^d^	0^d^	57.6^d^	115.2^ef^	492.4^ef^	62.8^ef^
	2226.5	1660.7	15.7	806.8^d^	0^d^	151.9^d^	120.4^ef^	178.1^ef^	141.4^ef^
	1100.1	3017.6	193.8	560.5^d^	0^d^	78.5^d^	47.1^ef^	220.0^ef^	104.78^ef^

Note: a is p < 0.001 compared with the young control group; b is p < 0.001 compared with the model control group; c is p < 0.001 compared with the treatment group. d is p<0.05 compared with the treatment group; e is p>0.05 compared with the model control group; f is p<0.01 compared with the treatment group.

### Thymus immunohistochemical staining

### Changes in P53, P16, SOD1, Sirt1 and Sirt3 expression in the thymus after mUCMSC treatment

**Figure 14:**
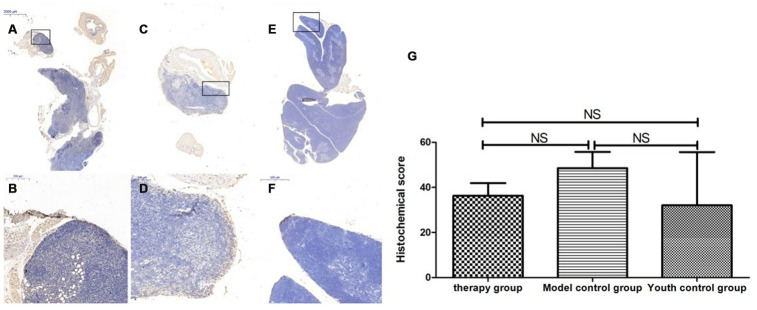
**Differences in P53 expression in mouse thymus tissue between different groups after mUCMSC treatment.** Note: (**A**, **B**) show immunohistochemical staining of p53 in the thymus tissue in the treatment group after treatment for 1 month. The expression of p53 protein in the thymus of the treatment group was increased. (**C**, **D**) show P53 immunohistochemical staining of mouse thymus tissue in the model control group. (**E**, **F**) show immunohistochemical staining of thymus P53 in young control mice. (**G**) shows the statistical analysis of histochemical scores. All dark brown tissue sections were strongly positive, brownish-yellow staining was moderately positive, light yellow staining was weakly positive, and blue nuclei were negative. *** indicates p < 0.001, ** indicates p < 0.01, * indicates p < 0.05, and NS indicates p > 0.05.

**Figure 15:**
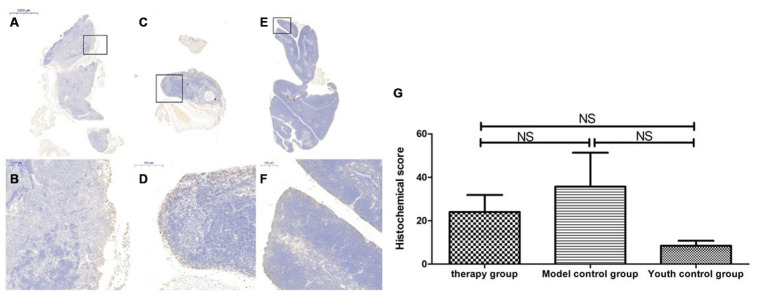
**Changes in p16 expression in the mouse thymus after treatment with mUCMSCs.** Note: (**A**, **B**) show P16 immunohistochemical staining of thymus tissue in the treatment group after treatment for 1 month. The expression of p16 protein in the thymus of the treatment group was increased. (**C**, **D**) show P16 immunohistochemical staining of mouse thymus tissue in the model control group. (**E**, **F**) show immunohistochemical staining of thymus P16 in young control mice. (**G**) shows the difference in P16 expression in mouse thymus tissue between different groups after mUCMSC treatment. All dark brown tissue sections were strongly positive, brownish-yellow staining was moderately positive, light yellow staining was weakly positive, and blue nuclei were negative. Note *** indicates p < 0.001, ** indicates p < 0.01, * indicates p < 0.05, and NS indicates p > 0.05.

**Figure 16:**
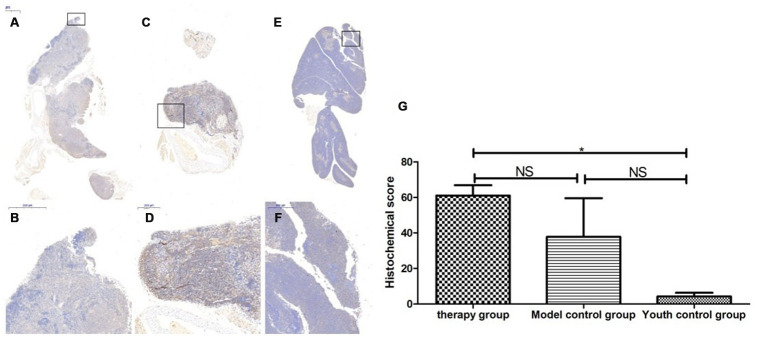
**SOD expression level in the mouse thymus after treatment with mUCMSCs.** Note: (**A**, **B**) show immunohistochemical staining of SOD in thymus tissue after treatment for 1 month in the treatment group. The levels of SOD1 were increased to different degrees in the treatment group. (**C**, **D**) show immunohistochemical staining of SOD in the mouse thymus tissue of the model control group. (**E**, **F**) show immunohistochemical staining of thymus SOD in young control mice. (**G**) shows the difference in SOD expression in mouse thymus tissue between different groups after mUCMSC treatment. All dark brown tissue sections were strongly positive, brownish-yellow staining was moderately positive, light yellow staining was weakly positive, and blue nuclei were negative. *** indicates p < 0.001, ** indicates p < 0.01, * indicates p < 0.05, and NS indicates p > 0.05.

**Figure 17:**
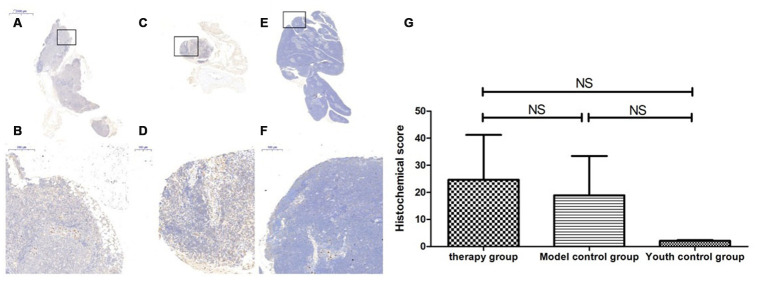
**Sirt1 expression level in the mouse thymus after treatment with mUCMSCs.** Note: (**A**, **B**) show immunohistochemical staining of Sirt1 in the thymus tissue after 1 month of treatment in the treatment group. The levels of Sirt1 were increased to different degrees in the treatment group. (**C**, **D**) show Sirt1 immunohistochemical staining of the mouse thymus tissue in the model control group. (**E**, **F**) show immunohistochemical staining of thymus Sirt1 in young control mice. (**G**) shows the difference in Sirt1 expression in mouse thymus tissue between different groups after mUCMSC treatment. All dark brown tissue sections were strongly positive, brownish-yellow staining was moderately positive, pale yellow staining was weakly positive, and blue nuclei were negative. *** indicates p < 0.001, ** indicates p < 0.01, * indicates p < 0.05, and NS indicates p > 0.05.

**Figure 18:**
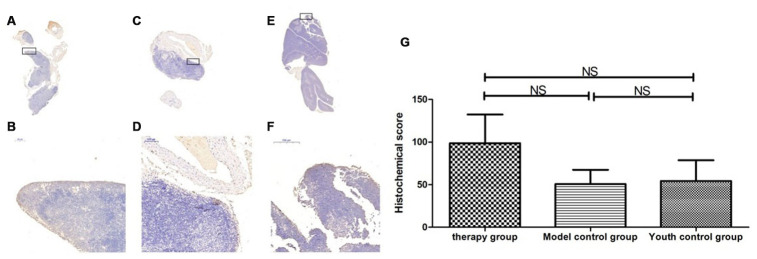
**Expression of Sirt3 in the mouse thymus after treatment with mUCMSCs.** Note: (**A**, **B**) show Sirt3 immunohistochemical staining of the thymus tissue in the treatment group after 1 month of treatment. The levels of Sirt3 were increased to different degrees in the treatment group. (**C**, **D**) show Sirt3 immunohistochemical staining of the mouse thymus tissue in the model control group; (**E**, **F**) show immunohistochemical staining of thymus Sirt1 in young control mice. (**G**) shows the difference in Sirt3 expression in mouse thymus tissue between different groups after mUCMSC treatment. All dark brown tissue sections were strongly positive, brownish-yellow staining was moderately positive, light yellow staining was weakly positive, and blue nuclei were negative. *** indicates p < 0.001, ** indicates p < 0.01, * indicates p < 0.05, and NS indicates p > 0.05.

The expression of p53 and P16 protein in the thymus of the treatment group was increased, but the difference was not significant (p>0.05) (Figures 14, 15, Tables 6, 7). The levels of SOD1, Sirt1, Sirt3 and other proteins with anti-oxidative stress and anti-aging functions were increased to different degrees in the treatment group (p>0.05) (Figures 16–18, Tables 8–10). This finding indicated that mUCMSC treatment has a certain degree of anti-aging activity.

**Table 6:** Comparison of the P53 expression levels among the treatment group, the model control group and the young control group after mUCMSC treatment (n=9).

**P53 expression level**	**therapy group**			**Model control group ^a^**			**Youth control group ^bc^**		
Number	1	2	3	1	2	3	1	2	3
Histochemical score (H-SCORE)	29.35	47.42	32	46.50	61.85	37.22	2.58	14.92	78.71

Note: a is p>0.05 compared with the treatment group; b is p>0.05 compared with the treatment group; c is p>0.05 compared with the model control group.

**Table 7:** Comparison of the P16 expression levels among the treatment group, the model control group and the young control group after mUCMSC treatment (n=9).

**P16 expression level**	**Therapy group**			**Model control group ^a^**			**Youth control group ^bc^**		
Number	1	2	3	1	2	3	1	2	3
Histochemical score (H-SCORE)	39.38	19.29	13.46	34.07	63.50	9.66	8.45	12.48	4.44

Note: a is p>0.05 compared with the treatment group; b is p>0.05 compared with the treatment group; c is p>0.05 compared with the model control group.

**Table 8:** Comparison of the SOD1 expression levels among the treatment group, the model control group and the young control group on the 28^t^^h^ day after the last cell transplantation (n=9).

**SOD1 expression level**	**Therapy group**			**Model control group ^a^**			**Youth control group ^bc^**		
Number	1	2	3	1	2	3	1	2	3
Histochemical score (H-SCORE)	58.6	52.54	72.1	77.2	34.00	2.26	3.80	0.90	8.13

Note: a is p>0.05 compared with the treatment group; b is p<0.05 compared with the treatment group; c is p>0.05 compared with the model control group.

**Table 9:** Comparison of the Sirt1 expression levels among the treatment group, the model control group and the young control group after mUCMSC treatment (n=9).

**Sirt1 expression level**	**Therapy group**			**Model control group ^a^**			**Youth control group ^bc^**		
Number	1	2	3	1	2	3	1	2	3
Histochemical score (H-SCORE)	57.7	7.34	8.92	47.8	5.91	3.11	1.64	2.42	2.23

Note: a is p>0.05 compared with the young control group; b is p>0.05 compared with the young control group; c is p>0.05 compared with the treatment group.

**Table 10:** Comparison of the Sirt3 expression levels among the treatment group, the model control group and the young control group after mUCMSC treatment (n=9).

**Sirt3 expression level**	**Therapy group**			**Model control group ^a^**			**Youth control group ^bc^**		
Number	1	2	3	1	2	3	1	2	3
Histochemical score (H-SCORE)	52.37	79.71	164.06	24.9	81.91	45.36	43.0	18.84	101.1

Note: a is p>0.05 compared with the treatment group; b is p>0.05 compared with the treatment group; c is p>0.05 compared with the model control group.

### Changes in the expression of LC3b, becline1 and P62 in the thymus after treatment with mUCMSCs

**Figure 19:**
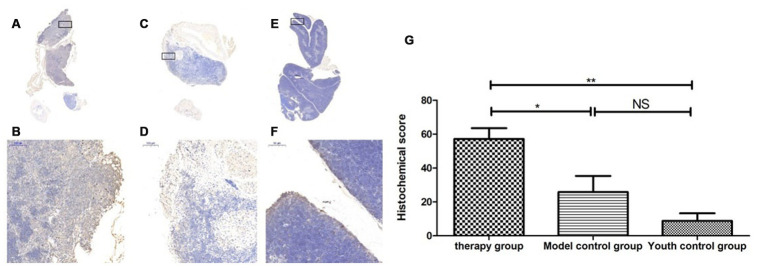
**Expression of LC3 in the mouse thymus after treatment with mUCMSCs.** Note: (**A**, **B**) show LC3 immunohistochemical staining of the thymus tissue after treatment in the treatment group. The levels of LC3 were increased to different degrees in the treatment group. (**C**, **D**) show LC3 immunohistochemical staining of the mouse thymus tissue in the model control group. (**E**, **F**) show immunohistochemical staining of thymus LC3 in young control mice. (**G**) Shows the difference in Lc3 expression in mouse thymus tissue between different groups after treatment with mUCMSCs. All dark brown tissue sections were strongly positive, brownish-yellow staining was moderately positive, light yellow staining was weakly positive, and blue nuclei were negative. *** indicates p < 0.001, ** indicates p < 0.01, * indicates p < 0.05, and NS indicates p > 0.05.

**Figure 20:**
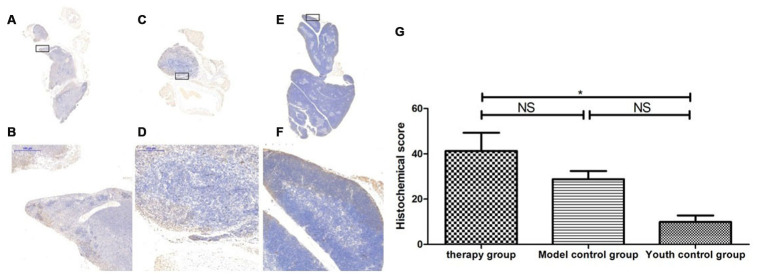
**Expression of becline1 in the mouse thymus after treatment with mUCMSCs.** Note: (**A**, **B**) show immunohistochemical staining of thymus becline1 in young control mice. (**C**, **D**) show immunohistochemical staining of thymus tissue becline1 in the treatment group after treatment for 1 month. (**E**, **F**) show immunohistochemical staining of becline1 in the mouse thymus tissue of the model control group. (**G**) shows the difference in the expression of becline1 in mouse thymus tissue between different groups after mUCMSC treatment. All dark brown tissue sections were strongly positive, brownish-yellow staining was moderately positive, pale yellow staining was weakly positive, and blue nuclei were negative. *** indicates p < 0.001, ** indicates p < 0.01, * indicates p < 0.05, and NS indicates p > 0.05. Becline1 activity was lowest in the young control group and highest in the treatment group, while the model control group showed intermediate activity.

**Figure 21:**
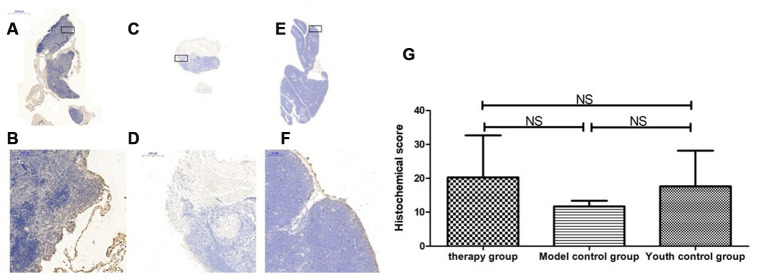
**Expression of P62 in the thymus tissue of mice after mUCMSC treatment.** Note: (**A**, **B**) show immunohistochemical staining of thymus P62 in young control mice. (**C**, **D**) show immunohistochemical staining of P62 in the thymus tissue after treatment for 1 month in the treatment group. (**E**, **F**) show immunohistochemical staining of P62 in the thymus tissue of model control mice. (**G**) shows the difference in P62 expression in mouse thymus tissue between different groups after mUCMSC treatment. All dark brown tissue sections were strongly positive, brownish-yellow staining was moderately positive, light yellow staining was weakly positive, and blue nuclei were negative. *** indicates p < 0.001, ** indicates p < 0.01, * indicates p < 0.05, and NS indicates p > 0.05. The P62 activity in the model control group was the lowest, and the P62 activity in the treatment group was the highest.

By comparing the levels of LC3b, becline1, P62 and other proteins that characterize the level of autophagy in the thymus, we found that the expression of LC3b, becline1, and P62 in the thymus tissue of the treatment group was increased to different degrees compared with that in the other groups. The LC3b activity was lowest in the young control group and highest in the treatment group, and the model control group showed intermediate activity (Figure 19, Table 11) (young control group/treatment group = 15.4% (p<0.01); young control group/model control group = 34.3% (p<0.05); model control group/treatment group = 45.1% (p>0.05)). Becline1 activity in the young control group was the lowest, and it was highest in the treatment group, while the model control group showed intermediate activity (Figure 20, Table 12) (young control group/treatment group = 24.0% (p<0.05); young control group/model control group = 34.4% (p<0.05); model control group/treatment group = 69.8% (p>0.05)). P62 activity in the model control group was the lowest, and P62 activity in the treatment group was the highest; P62 activity was intermediate in the young control group (Figure 21, Table 13) (young control group/treatment group=87.0% (p>0.05); young control group/model control group=150.6% (p>0.05); model control group/treatment group=57.7% (p>0.05)).

**Table 11:** Comparison of the LC3 expression levels among the treatment group, the model control group and the young control group after mUCMSC treatment (n=9).

**LC3 expression level**	**Therapy group**			**Model control group^a^**			**Youth control group^bc^**		
Number	1	2	3	1	2	3	1	2	3
Histochemical score (H-SCORE)	48.0	54.0	69.5	8.25	41.1	28.0	1.71	7.83	17.0

Note: a is p<0.05 compared with the treatment group; b is p<0.01 compared with the treatment group; c is p>0.05 compared with the model control group.

**Table 12:** Comparison of the expression levels of becline1 among the treatment group, the model control group and the young control group after mUCMSC treatment (n=9).

**Becline1 expression level**	**Therapy group**			**Model control group^a^**			**Youth control group^bc^**		
Number	1	2	3	1	2	3	1	2	3
Histochemical score (H-SCORE)	55.7	27.80	40.16	35.96	26.24	24.24	5.63	8.76	15.35

Note: a is p>0.05 compared with the treatment group; b is p<0.05 compared with the treatment group; c is p>0.05 compared with the model control group.

**Table 13:** Comparison of the P62 expression levels among the treatment group, the model control group and the young control group on the 28^th^ day after the last cell transplantation (n=9).

**P62 expression level**	**Therapy group**			**Model control group^a^**			**Youth control group^bc^**		
Number	1	2	3	1	2	3	1	2	3
Histochemical score (H-SCORE)	44.98	5.92	9.81	11.75	8.75	14.57	2.67	37.98	12.18

Note: a is p>0.05 compared with the treatment group; b is p>0.05 compared with the treatment group; c is p>0.05 compared with the model control group.

### Changes in thymocyte subsets after mUCMSC treatment

**Figure 22:**
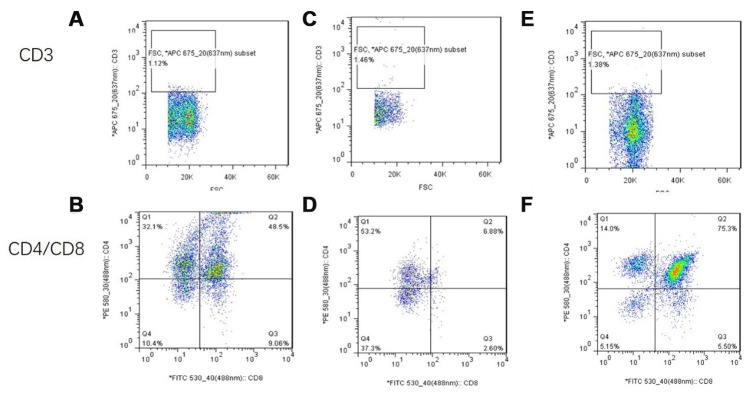
**Flow cytometry analysis of single-cell suspensions of thymus tissue after mUCMSC treatment.** Note: (**A**, **B**) show flow cytometry of thymocytes in the treatment group after 1 month of treatment. (**C**, **D**) show flow cytometry of a single-cell suspension of mouse thymus tissue from the model control group. (**E**, **F**) show flow cytometry detection of a single-cell suspension of thymus tissue from the young control group. CD3 was not expressed in any of the three groups. The proportion of CD8+ cells in the treatment group was significantly higher than that in the model control group.

CD3 was not expressed in any of the three groups. The proportion of CD8+ cells in the treatment group was significantly higher than that in the model control group (Figure 22).

## DISCUSSION

### Aging model evaluation

Aging is a process in which the tissue and organ structure of an organism is spontaneously degenerated, with functional degradation and reduced overall adaptability. Aging is a major risk factor for the development of chronic diseases affecting various tissues, such as the cardiovascular system, immune system, muscles and bones [14]. With the increase in the aging population in China, delaying aging has become a hot topic at home and abroad. The establishment of a reliable animal model of aging has become an essential problem to be solved in the study of aging. At present, artificial induction of aging is mainly carried out by D-galactose injection and ozone-induced damage. The theoretical basis for establishing this aging model is mainly based on the current theory that states that metabolism and free radicals underlie the mechanism of aging. However, this theory cannot completely explain biological aging. Thus, there are considerable limitations to this theory. To better represent the characteristics of natural aging of the human body, this study used animals that were naturally aged to establish an aging model to obtain more realistic experimental results.

C57 mice are a commonly used animal model with stable and easy breeding characteristics. C57 mice have early maturity and strong fertility. Generally, females are mature when they are 35-50 days old, and males are mature when they are 45-60 days old; female mice have sexual cycles lasting 4-5 days, and gestation is 19-21 days. The life expectancy of C57 mice is approximately 2-3 years. C57 mice are often used in aging research worldwide. This study used naturally aging C57 mice as an aging model. According to the mouse-to-human age exchange ratio, 2 years old in mice is equivalent to 70 years old in humans, and more than 50% of the natural life expectancy is 2 years, so 1.5 years old was selected as the age for MSC transplantation.

In this experiment, C57 mice were purchased and maintained in the SPF animal laboratory at our hospital for 18 months. According to the life cycle of C57 mice, the mice in the group were in the old age stage of their life cycle. Their appearance was characterized by partially white hair, loss of back hair, local alopecia areata, laxity of movement, poor mental health, reduced activity, and body weight in the range of 38±5 g. Structural observation showed the presence of thymus gland atrophy and disappearance of the medullary gland-like structure in 18-month-old mice. Immunohistochemical staining analysis showed that the protein expression of p53 and P16 increased and the protein expression of SOD1, Sirt1 and Sirt3 decreased. Therefore, the above detection indicators can confirm that the aging model established in this study is consistent with the characteristics of aging at the old age stage, including overall appearance and molecular changes.

### Evaluation of the therapeutic effect of mUCMSCs

MSCs are stem cells that can differentiate into different germ layer tissues in a specific environment. The body has specific mechanism for the repair of tissue and organ damage and the regulation of immune responses. Compared with bone marrow and adipose-derived MSCs, UCMSCs have the advantages of convenient access, good survival in the host and low immunogenicity [15]. They are expected to be applied in alleviating transplantation immune rejection, tissue engineering, anti-aging, and medical beauty procedures. However, more research is needed for the large-scale clinical application of MSCs at this stage.

In this study, the umbilical cord was obtained from C57 mice at 21 days of pregnancy to ensure that the umbilical cord was long enough to facilitate the primary culture of mouse UCMSCs. The cells were adherent in the culture flask, and the cells were long and fusiform. They had the potential to differentiate into fat cells, chondrocytes, and bone cells and were subcultured for third-generation cryopreservation, providing a cellular basis for research.

This study compared the control group of mice from the same stage of the lifecycle and verified that mUCMSCs have the function of regenerating the thymus structure in aged C57 mice. (1) The thymus volume of the C57 mice in the treatment group was significantly larger than that in the model control group. The thymus index was calculated and statistically analyzed. The mean value of the thymus index was compared (model control group/treatment group = 0.15, p<0.01). However, according to HE staining, the thymus tissue structure of the treatment group was significantly restored compared with that of the model control group. The thymus cortex structure of the model control group was unclear with respect to quality, and no glandular structure was observed. (2) The thymic medulla and cortex were labeled with the keratin antibodies ck5 and ck8, respectively. The statistical analysis showed that the cumulative optical density (IOD) of CK5 expression in the thymic medulla of the model control group was higher than that of the young group (p< 0.01). This result indicated that the density of epithelial stromal cells expressing CK5 in the medulla was increased, the proportion of epithelial stromal cells was increased, and the proportion of lymphocytes that did not express CK5 was decreased. This suggested that the size of the thymic medulla in C57 mice increases with age, the proportion of lymphocytes decreases, and the proportion of thymic medulla stromal cells increases. The expression level of CK5 in the thymus of the treatment group was lower than that of the model control group (p<0.01), indicating that the proportion of lymphocytes that do not express CK5 in the medulla of the senile mice increased after mUCMSC treatment, and the total number of cells as well as the thymus volume increased. CD4 and CD8 immunofluorescence staining revealed a thymic cortex with lymphocytosis. This indicates an increase in the number of lymphocytes that accumulated in the medulla. This suggested that mUCMSC treatment can promote lymphocyte homing to the medulla of aged mice or promote their proliferation in the medulla. The expression of CK8 in the thymic cortex of the model control group and the treatment group was higher than that of the young group. This suggested that the proportion of thymic cortex stromal cells expressing CK8 increases with age, and the proportion of lymphocytes in this region decreases. Compared with that in the model control group, the thymus CK8 expression in the treatment group was higher than that in the model control group (p>0.05), suggesting that the proportion of thymic cortical stromal cells after treatment of elderly mice with mUCMSCs was not significantly changed. The ability of mUCMSC treatment to increase the proportion of lymphocytes in the thymic cortex of aged mice was not obvious. In summary, mUCMSCs can improve senile sensitization, mainly by increasing the proportion and number of lymphocytes in the medulla of mice. mUCMSC treatment can promote lymphocyte homing to the medulla of aged mice or promote proliferation in the medulla region.

### Possible mechanism of mUCMSC efficacy

How do mUCMSCs regenerate the thymus structure of aged C57 mice? A literature review showed that the percentages of lymphocyte subpopulations vary with age. The evidence shows that the changes from childhood to adulthood proceed as follows: the CD4^+^/CD8^+^ ratio decreases with age, which is mainly due to the rapid increase in the number of CD8^+^ cells, and the CD4^+^/CD8^+^ ratio in adulthood increases. This shows that young children and seniors show an immune imbalance. In the former, this reflects immune immaturity, and in the latter, this reflects weakened immunosuppressive function.

Therefore, we asked whether mUCMSCs also change the proportions of different subpopulations of lymphocytes in the thymus. In this study, fluorescence staining with CD4 and CD8 antibodies revealed that the number of CD4^+^ T and CD8^+^ T cells in the treated group was significantly higher than that in the model control group, and the increase in CD8^+^ T cells was the most obvious. The change in the mUCMSC transplantation group compared with the model control group indicated that mUCMSC transplantation can restore the distribution of lymphocyte subsets from the old age state to the youth state. Intact thymus tissue was digested with type II collagenase into a single-cell suspension. The proportions of the T lymphocyte subsets were analyzed by flow cytometry, which showed that the number of CD4^+^ T and CD8^+^ T cells was increased significantly in the treated group compared with the model control group. The most obvious increase was observed in CD8^+^ T cells. The results obtained by fluorescence staining of CD4 and CD8 antibodies were consistent. Therefore, we asked whether these changes suggest that the mouse thymus tissue changes to a young state after mUCMSC transplantation.

By immunohistochemical detection of P53, P16, SOD1, Sirt1, Sirt3 and other aging proteins, we found that the expression of p53 and P16 protein in the thymus of the treated group increased (p>0.05). The levels of SOD1, Sirt1, Sirt3 and other proteins with anti-oxidative stress and anti-aging functions increased to different degrees in the treatment group (p>0.05). This suggests that the degree of aging in tissues is reduced, indicating that treatment with mUCMSCs can confer a certain degree of anti-aging activity. p53 can cause endogenous apoptosis by activating proapoptotic genes [16] or by inhibiting the expression of the anti-apoptotic genes BCL-2 and BCL-xL [17]. mUCMSCs inhibit the occurrence of endogenous apoptosis by downregulating the expression of the p53 gene.

By comparing LC3b, becline1, P62 and other proteins that characterize the level of autophagy in the thymus, we found that the expression of LC3b, becline1 and P62 in the thymus tissue of the treatment group was increased to different degrees compared with that in the other groups. The LC3b activity was the lowest in the young control group and was the highest in the treatment group, and the model control group showed intermediate activity (Figure 19, Table 11) (young control group/treatment group = 15.4% (p<0.01); young control group/model control group = 34.3% (p<0.05); model control group/treatment group = 45.1% (p>0.05)). Becline1 activity in the young control group was the lowest, and it was the highest in the treatment group, while the model control group showed intermediate activity (Figure 20, Table 12) (young control group/treatment group = 24.0% (p<0.05); young control group/model control group = 34.4% (p<0.05); model control group/treatment group = 69.8% (p>0.05)). P62 activity in the model control group was the lowest, and P62 activity in the treatment group was the highest; P62 activity was intermediate in the young control group (Figure 21, Table 13) (young control group/treatment group=87.0% (p>0.05); young control group/model control group=150.6% (p>0.05); model control group/treatment group=57.7% (p>0.05)). The expression of these proteins, which represent the level of autophagy, in the treatment group was higher than that in the model control group. The therapeutic effect of mUCMSCs may be mediated by upregulating the expression of the LC3b, becline1, and P62 genes in the thymus, increasing the level of autophagy in the thymus tissue, and downregulating the level of apoptosis in cells, thereby stabilizing thymocytes and promoting thymic regeneration. Moreover, the expression levels of genes with anti-oxidative stress and anti-aging functions, such as SOD1, Sirt1 and Sirt3, were upregulated, and the expression of the p53 and P16 genes was decreased, which promoted the recovery and regeneration of the thymus.

## MATERIALS AND METHODS

### Screening of the natural aging model in C57 mice

Fifty 8-day-old SPF-class C57BL/6 female mice were purchased. They were bred in the SPF animal laboratory of the 920^th^ Hospital of the People's Liberation Army Joint Logistics Support Force. After 18 months of age, the mice showed white hair, slow movement, poor mental activity, and reduced activity, and a weight range of 38±5 g was selected as the standard. Mice were selected, and 3 mice were randomly weighed and sacrificed by cervical dislocation. The thymus was dissected, and the thymus index was compared with the thymus index of 2-month-old mice. The old C57 mouse thymus index/young C57 mouse thymus index was 8.4%. Fixation with 4% paraformaldehyde, paraffin embedding, pathological sectioning, and HE staining showed evidence of thymic gland atrophy and structural disorder, and immunohistochemistry was used to detect the expression of aging-related proteins. It is suggested that the aging model was established successfully. Thirty female mice that met the criteria were randomly selected and included in the experiment.

### Isolation and culture of C57 mouse mUCMSCs

Pregnant C57 mice, which were raised in the SPF animal laboratory of the 920^th^ Hospital of the People's Liberation Army Joint Logistics Support Force and were pregnant for 21 days, were sacrificed by cervical dislocation; the umbilical cord was aseptically collected, and the tissue block was cut into 1 mm^3^ tissue sections. The blocks were inoculated on the bottom wall of a 10 cm^2^ culture dish. After MSCs migrated and grew at a fusion rate of 80%, the primary mUCMSCs were digested with trypsin and further expanded and cultured to the third generation (P3).

### Identification of C57 mouse mUCMSCs

For the P4 generation mUCMSCs, the growth morphology during culture was observed under an inverted microscope. The positive expression rates of CD29, CD90 and CD34 in mUCMSCs were analyzed by flow cytometry. Using the induced differentiation assay kit, adipogenic, osteogenic and chondrogenic differentiation was performed according to the instructions of the manual, and the differentiation potential was identified.

### Grouping and processing of experimental animals

The C57 mice that met the requirements of thymic aging in the previous screening were randomly divided into the treatment group and the model control group. At the same time, 2-month-old C57 mice were used as the youth control group, with 15 mice in each group. They were all kept in the SPF animal laboratory.

### Tail vein transplantation of mUCMSCs

For the experimental group, P4 generation mUCMSCs were infused through the tail vein at a dose of 1×10^7^ cells/kg twice a week (Monday and Thursday) for 3 weeks; the other 2 groups were injected with an equal volume of normal saline. After routine feeding and daily recording of mouse activity, the mice were sacrificed by cervical vertebra dislocation 6 weeks after the last transplantation, and the efficacy and mechanism were evaluated.

### Transplantation efficacy evaluations

### Changes in coat color, thymus morphology and size and the thymus index in C57 mice

After assigning the mice to different groups, images were obtained under the same lighting conditions, and the average grayscale value of the head, back and hip in the photos was measured and tested by one-way ANOVA. The thymus specimens were arranged in groups and photographed. The body weight of each mouse and the corresponding thymus weight were recorded. The thymus index was calculated, and one-way ANOVA was performed.

### Observation of the thymus structure in C57 mice

C57 mice were sacrificed by cervical dislocation. The abdominal cavity was cut open and cut along the rib cartilage on both sides to the upper edge of the thoracic spine. The thymus was exposed. After weighing and comparing the photos separately, the thymus was fixed with 4% paraformaldehyde. The thymus tissue was collected, embedded in paraffin, sectioned, and stained with HE. The changes in the tissue structure were observed under a light microscope.

### Histological observation

### Staining and observation of CK8, CK5, CD4 and CD8 immunofluorescent antibodies in C57 mice

A portion of the thymus was fixed with 4% paraformaldehyde, embedded in paraffin, sliced, and subjected to immunofluorescence staining. The green/red fluorescent nuclei with the same labeling were selected by ImagePro Plus 6.0 software as the unified standard for judging all photopositive cells, and each photo was analyzed to obtain the number of positive cells in each field of view (approximately 0.190875 mm^2^). The positive cell density (n/mm^2^) was determined. The expression of CK8, CK5, CD4, and CD8 in the thymus was scored.

### Immunohistochemical staining and observation of the thymus in C57 mice

A portion of the thymus was fixed with 4% paraformaldehyde, embedded in paraffin, and sectioned, and immunohistochemical staining was performed. The analysis software with Pannoramic viewer was used after the image scan was completed. The densito quant software in the Quant center automatically recognizes and determines all the dark brown staining in the tissue section to be strongly positive, the brownish-yellow staining to be moderately positive, the pale yellow staining to be weakly positive, and the blue nuclei to be negative. Furthermore, for each tissue, the areas of strongly positive, moderately positive, weakly positive and negative staining, the percentage of positive staining, and the final H-score were determined in units of pixels. P53, P16, SOD1, Sirt1, Sirt3, LC3b, becline1, and P62 expression changes were observed and evaluated in the mouse thymus.

### Proportional analysis of immune cell subsets in thymus tissue

The thymus tissue was cut into small 1 mm^3^ tissue pieces with scissors and digested with 0.1% type II collagenase at 37 °C for 30 mins. The cells were filtered through a 70-mesh filter, and the cells were observed to show a single state under a microscope. The cells were centrifuged at 400×g for 5 mins and then resuspended in 1× PBS to a concentration of 1×10^7^ cells/mL, after which 100 μL of cell suspension was added per tube with 5 μL each of anti-mouse PE-CD4, FITC-CD8, APC-CD3, PE-CF594-FOXP3, and Percp-cyTm5.5-CD25 antibodies; equal amounts of PE-IgG, FITC-IgG, APC-IgG, PE-CF594-IgG, and Percp-cyTm5.5-IgG were added to the isotype control tube. The cells were incubated at room temperature for 30 mins in the dark, and then 300 μL of PBS was added, after which the cells were filtered with a 400-mesh filter before detection. Detection was performed using a BD FACSAria III flow cytometer and analyzed using FlowJo 7.6 software. Changes in mouse thymus lymphocyte subsets were observed.

### Statistical analyses

Statistical analysis was performed using SPSS 24.0 software. Measurement data were expressed as the mean ± standard deviation (x ± s), and independent samples were analyzed by t tests. Statistical comparisons between groups were tested by one-way ANOVA. P < 0.05 indicated a significant difference.

## CONCLUSIONS

The C57 mouse model with thymic atrophy showed partial whitening, shedding, alopecia areata, slow movement, decreased activity, atrophy of the thymic gland, disappearance of the medullary gland-like structure, and increased expression of the p53 and P16 genes.

C57 mouse mUCMSCs with different growth characteristics, immunophenotypes and differentiation potential in accordance with UCMSC standards were prepared.

After treatment with mUCMSCs, the thymus volume of the mice increased, the weight increased, parts of the thymic structure regenerated, the number of lymphocytes in the thymic medulla increased, and the functioning of CD8+ T cells improved.

mUCMSCs inhibit the expression of the aging-related genes p53 and P16 by promoting the expression of autophagy- and anti-oxidative stress-related genes, such as LC3b, becline1, P62, SOD1, Sirt1 and Sirt3, thereby increasing the level of autophagy in the thymus tissue, inhibiting apoptosis and exerting therapeutic effects on thymus atrophy.

### Ethics approval

The experimental protocols were approved by the Experimental Animal Ethics Committee of the 920^th^ Hospital of the PLA Joint Logistics Support Force.
